# Black Soldier Fly Culture as a Source of Chitin and Chitosan for Its Potential Use in Concrete: An Overview

**DOI:** 10.3390/polym17060717

**Published:** 2025-03-08

**Authors:** Hugo González-Lara, Benito Parra-Pacheco, Enrique Rico-García, Humberto Aguirre-Becerra, Ana Angélica Feregrino-Pérez, Juan Fernando García-Trejo

**Affiliations:** 1 División de Investigación y Posgrado, Facultad de Ingeniería, Universidad Autónoma de Querétaro, Carretera a Chichimequillas Km. 1 s/n, Amazcala, El Marqués 76265, Querétaro, Mexico; laragonhugo@gmail.com (H.G.-L.); benito.parra@uaq.mx (B.P.-P.); 2 Cuerpo Académico de Bioingeniería Básica y Aplicada, Facultad de Ingeniería, Universidad Autónoma de Querétaro, Cerro de las Campanas s/n, Las Campanas, Santiago de Querétaro 76010, Querétaro, Mexico; ricog@uaq.mx (E.R.-G.); humberto.aguirre@uaq.mx (H.A.-B.); geli@uaq.mx (A.A.F.-P.)

**Keywords:** biopolymer, entoconcrete, green concrete, organic waste, sustainability

## Abstract

Chitin is one of the most abundant biopolymers in nature and is found mainly in the exoskeletons of crustaceans and insects, in the cell walls of fungi, and in some species of mollusks. Chitosan is a derivative of chitin; it is much more accessible and has a broader range of applications, including improving the quality of materials such as films, plastics, and concrete. The rheological properties of chitin and chitosan refer to their behavior against deformation and flow and their ability to resist structural changes under mechanical stress conditions. These properties are fundamental in applications where the aim is to control the texture, viscosity, and handling of these biopolymers. Three types of methods for the extraction of chitin and chitosan can be classified: the first is the chemical method, which presents high yields but uses reagents that generate toxic residues; the second is the biological method, which takes advantage of chemical reactions of microorganisms but in some cases has low yields compared to chemical extraction; and the third is the enzymatic method, which uses reagents with a low production of toxic residues. However, low extraction yields are also reported. One of the primary sources of chitin and chitosan is the residue of shellfish and crustaceans. However, a new source of obtaining these compounds is the black soldier fly, which has the same yields of biopolymers as shellfish. In addition, this is a residue of the black soldier fly larvae culture, where protein, oil, and biofertilizers are generated by the bioconversion of organic waste. This work proposes the black soldier fly as an alternative source for extracting chitin and chitosan, using organic methodologies that do not generate toxic residues and have high yields. Including these biopolymers in concrete elaboration could have positive results in terms of flexibility, compressive strength, and workability.

## 1. Introduction

Concrete is one of the most used materials in construction due to its structural properties such as compressive and flexural strength. It is a mixture of crushed stone, sand, water, and cement. The main application of concrete is for the formation of foundation elements, columns, beams, load-bearing walls, and slabs; building blocks and some other non-structural elements are also made for architectural purposes. Cracking is one of the main causes of concrete deterioration. During the first 8 h after casting, in its plastic state, the concrete suffers cracking due to volumetric change, evaporation, and capillary pressure [1]. In the second state, after hardening, cracking can be caused by freeze–thaw damage, creep deformation, chemical distress, thermal shrinkage, and overloading [2]. Adding steel, glass, and carbon-based polymers is the most effective method to increase compressive and flexural strength and reduce concrete cracking [3].

The accelerated growth of the construction industry has highlighted for engineers the sustainability and use of environmentally friendly materials. Biopolymers, such as chitin and chitosan, have been used as an additive due to the structural properties that they provide [4].

Chitin and chitosan are two of the most common biopolymers in nature. There are natural sources of this material such as ringed worms, insects, yeasts, fungi, and some algae [5]. The most relevant uses of chitin are as a growth promoter in plants by hormone activity [6] and as a wound dressing to promote skin regeneration [7]. Chitosan has been used as mucoadhesive [8], antitumoral [9], anti-inflammatory [10], antioxidant [11], antimicrobial [12], and antifungal material [13]. Chitosan is more commonly used than chitin, due to the protein links it forms, which makes it a somewhat unstable structure. Therefore, demineralization and deproteinization are necessary for chitin purification [14]. The main sources of chitin and chitosan are shrimp and crabs [15]; however, black soldier fly is an emergent new source [16].

The increase in black soldier fly biomass production for protein extraction will provide a high amount of insect chitin and chitosan [17]. The black soldier fly is presented as a sustainable alternative for obtaining chitin due to its efficient rearing in small spaces, low resource consumption, and feeding on organic waste. This reduces the environmental impact compared to other sources of chitin and contributes to the circular economy by taking advantage of organic waste [18]. This work aims to describe the properties of chitin and chitosan, their extraction from black soldier fly, and the effects of these biopolymers on concrete.

## 2. Chemical Description of Chitin and Chitosan

Chitin is a linear polymer composed of the amino sugar 1–4 linked 2-acetamido-2-deoxy-β-D-glucopyranose (C_8_H_13_O_5_N)n [19]. There are three allomorphic crystal forms, named α, β, and γ, that were identified from X-ray diffraction studies; the crystalline and molecular order of chitin involves the physiological function and the characteristics of the tissue of chitin [20]. α-chitin could be found in crustacean shells, beetle, and fungi cell walls; it is the most stable crystalline form of chitin, with polymer chains arranged in an antiparallel way with strong hydrogen bonding [21]. β-Chitin is mostly found in marine diatoms, mollusks, and the peritrophic matrix in insects, and polymer chains are arranged in a parallel order, offering a more flexible open structure [22]. γ chitin is little known due to its limited presence in nature; however, it has been found in beetle cocoon fibers; the arrangement crystalline structure consists of three chains, with alternating parallel and antiparallel aligned polymers [23]. Chitin, by the chemical deacetylation reaction, can be transformed into chitosan. Chitosan is much easier to process than chitin, but the stability of chitosan materials is generally lower due to its more hydrophilic character and especially its sensitivity to pH [15].

**Figure 1:**
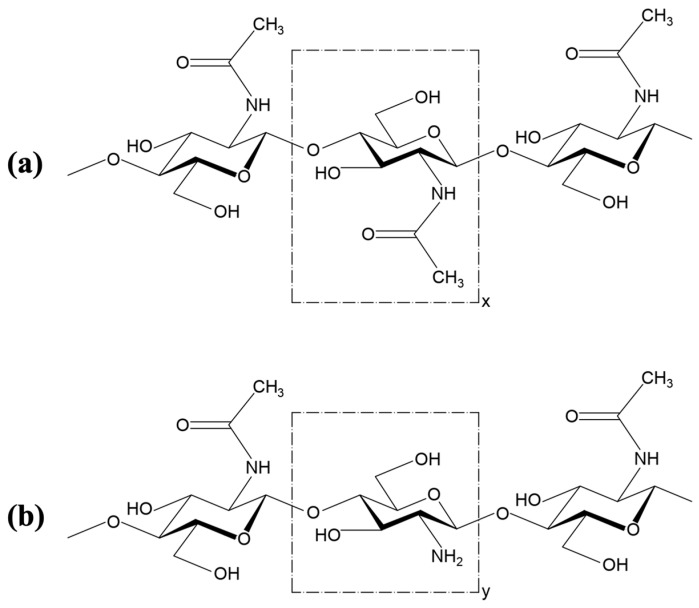
Chemical structure of (**a**) chitin, where “x” is the acetylated unit, and (**b**) chitosan, where “y” is the deacetylated unit.

Chitosan consists of reactive groups such as primary amino and primary and secondary hydroxyl, in addition to functional groups such as acetamide and glycosidic bonds, compared to its predecessor chitin. Figure 1 describes the chemical structure of chitin and chitosan. All these groups make chitosan a polymer with new properties and behaviors [24]. Both polymers have variability due to their natural origin, which makes it very difficult to compare the results and establish relationships between the physiological behavior of chitin and chitosan and their properties; these properties are mainly molecular weight and acetylation degree [25].

Chitin can form complexes with cholesterol, proteins, and peptides. It is insoluble in alcohols, alkalis, acids, water, and other organic solvents; this property reduces the possibility of its use in the materials industry. Among the various derivatives of this biopolymer, chitosan is the most accessible. Chitosan is soluble in dilute inorganic and some organic acids. This dependence of solubility on pH allows for the use of chitosan in various forms: nanoparticles, bio-nanocomposites, films, membranes, and fibers. The molecular structure of chitosan improves its physical, chemical, and physiological functions [26]. These properties have enabled chitin and chitosan to improve the quality of materials such as films, plastics, and concrete. There are works that report an increase in the properties of concrete with the use of both biopolymers; however, the advantages of using one or the other are not clear.

## 3. Rheological Properties of Chitin and Chitosan

The main rheological properties reported of chitin and chitosan are crystallinity index and degree of deacetylation; they can be influenced by the interactions among proteins, fibers, and starch molecules, which can be affected by processing procedures [27].

The crystallinity index can be used to determine the function of polymers; chitin with low values has absorbent properties, whereas chitin with high values can be used on nanofibrils; however, for chitosan, the crystal dimension decreases due to deacetylation because of the chemical treatment [28]. Crystallinity depends on the nature of the organism from which the chitin was isolated and the method employed in the extraction of the polymer [29]; insect chitin has similar values to shellfish, so it could be considered an alternative green source [30].

The degree of deacetylation is used to determine the type of polymer; for those with values higher than 50%, they are considered chitosan, and those with values less than 50% are chitin. This parameter directly influences their physicochemical and mechanical properties [31]. Triunfo et al. [28] reported that the chemical deacetylation process alters the initial structure of chitin from black soldier flies, producing a more homogeneous, rough, and less fibrillated chitosan. In insects, it has been observed that the degree of deacetylation varies according to the functions of the chitin in the body parts and the different phases of the insect life cycle; this can directly influence the chitin content and, consequently, the amount of chitosan obtained [29].

Chitin and chitosan are structural components in the cell wall of insects; this characteristic allows them to be used as additives in concrete [32]. They reduce the surface texture of the cement, making a thick mixture by the formation of agglomerates, offering greater elasticity and adhesion at the nano level with a positive effect at the macro level. They also have a high modulus and tensile strength, which increases their mechanical properties [33]. Additionally, they also give viscosity to the materials; this is due to the conformation and structural flexibility of their molecules, offering a higher viscosity when they have a higher degree of deacetylation [30].

## 4. Isolation of Chitin and Chitosan

On a large scale, the isolation method is essential because high yields are required, and the technique must be low-cost and have a minimum environmental impact without altering the physical and chemical properties of the biopolymers [34]. The extraction of chitin is usually divided into three stages: demineralization (DM), deproteinization (DP), and decolorization (DC); subsequently, the deacetylation process is executed by removing the acetyl group to transform it into chitosan. Isolation can be performed by chemical or bio-based methods.

### 4.1. Chemical

The main sources of extraction reported recently are shellfish and insects; to prepare the sample, it is dried at 105 °C for 48 h and subsequently ground. The first step is DM at a ratio of 1:10 mass/volume with hydrochloric acid (HCl) 1 M at room temperature for 1 h. Subsequently, DP is performed, and the sample is mixed with sodium hydroxide (NaCl) 1 M at a radio of 1:25 mass/volume for 1 h at 80 °C; this step is repeated 12 times until a bleached sample is obtained. Finally, the solids formed are removed by filtration, washed with distilled water, and dried at 105 °C for 48 h [35].

However, for DM, the use of other acids such as formic acid (HCOOH), citric acid (C_6_H_8_O_7_), nitric acid (HNO_3_), and sulfuric acid (H_2_SO_4_) has been reported, as well as different concentrations from 0.25 to 4 M. Generally, the reaction is carried out at room temperature; however, it has been performed at 100 °C spanning time frames from 10 min to 2 days. For DP, sodium carbonate (Na_2_CO_3_) and sodium phosphate (Na_3_PO_4_) have also been reported at concentration ranges from 0.25 to 4 M, using a higher temperature range, from 60 to 100 °C, for a period of 3 days or even just 15 min [14,36].

### 4.2. Bio-Based

#### 4.2.1. Biological

Some microorganisms have been used to remove mineral and protein contents to offer an alternative that does not generate toxic waste. For chitin DP from shrimp shell waste, *Halobacterium salinarum* and *Halococcus dombrowskii* have been used by fermentation [37]. Another study used *Bacillus subtilis* and *Acetobacter pasteurianus* to extract chitin from black soldier fly. Biological DP and DM obtained higher yields compared to the chemical methodology; however, deacetylation was lower [38]. On the other hand, lower yields of chitin have been reported using *Bacillus lichenformis* A6 [39].

#### 4.2.2. Enzymatic

Another alternative method is the use of enzymes for the extraction of chitin from small shrimp, as well as the use of alcalase for DP, in parallel increasing the degree of acetylation [40]. Enzymatic hydrolysis by a *Bacillus licheniformis* protease has been used for the extraction of black soldier fly chitin; however, the yields were lower than the chemical methods due to proteins not being separated efficiently [17].

**Figure 2:**
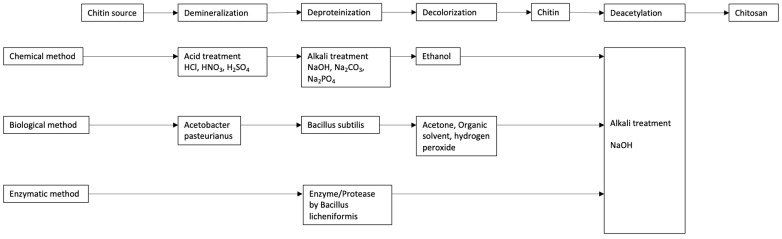
Chitin and chitosan isolation scheme by chemical, biological, and enzymatic methods.

The method for the extraction of chitosan influences its resulting physical and chemical properties. The biological and enzymatic extraction methods are less solvent and energy-intensive. However, their fundamental problem is that they are not effective at removing remaining protein and minerals on a commercial scale [14]. Chemical deacetylation is the most effective method; however, it has a high energy consumption, uses very corrosive materials, and generates toxic waste. Enzymatic deacetylation, using chitin deacetylase of fungal and bacterial origin, is a more environmentally friendly alternative due to its less aggressive and destructive nature; however, its efficiency varies [41]. Figure 2 schematically describes the inputs used in the chemical isolation and bio-based methods for extracting chitin and chitosan.

Table 1 compares the advantages and disadvantages of the chitin and chitosan extraction methods and provides a brief description of the process.

In terms of energy efficiency, reaction times are shorter with biotechnological methods than with conventional chemical methods, reducing energy consumption during deacetylation from 382.1 to 8.9 kJ per gram of chitosan [42].

## 5. Chitin and Chitosan Content in Black Soldier Fly

Due to their high chitin content, some aquatic organisms, insects, and fungi are the primary sources of chitin. According to some authors, the organisms with the highest chitin content are Lobster (*Nephro*) at 69.8% [43], Shrimp shell waste at 20% [21], silkworm (*Bombyx mori*) at 96.75%, Rhinoceros beetle (*Allomyrina dichotoma*) at 83.37% [36], and *Aspergillus niger* at 42% [44]. However, black soldier flies have been reported to contain higher percentages than previously mentioned organisms.

The black soldier fly larva is used to produce protein flour [45], oil derivatives [46], and for the bioconversion of organic waste [47]. During the breeding time of this insect, waste such as frass is produced, which is used as biofertilizer for plants [48]. Frass is made up of undigested waste, larva excreta, and fly parts at their different physiological stages. At all stages, from egg to adult, the fly is rich in chitin and chitosan; therefore, this residue can be considered an alternative source of these biopolymers [18].

It is estimated that the production of black soldier fly larvae is 300 billion larvae annually [16]. Considering an average larva weight of 212 milligrams fed with food waste [49], a production of 63 million tons of larva in fresh weight is estimated, so there is the opportunity to take advantage of the production of larva for the extraction of chitin or chitosan and put them to use.

Some works have been reported on the extraction and purification of chitin and chitosan. The method, reagents, and growth stage of the fly have a direct effect on the amount of chitin and chitosan that can be extracted, and even the type of feed residue of the larvae, since it has been described that prepupae fed with vegetable residue contain 11.76% chitin, and those fed with fruit residue contain less, at 6.82% [50].

Larvae of the fifth instar have a high lipid content. For the extraction of chitin and chitosan, the first step is defatting; secondly, dark pigmentation can be observed in the last instar of ontogenesis due to the melanin, covalently linked to chitin, being impossible to separate from the complex without destroying the chitin. Therefore, to obtain chitin and chitosan, it is necessary to use non-pigmented larvae. Subsequently, for the extraction of chitosan, the standard procedure of defatting, DM, DP, and deacetylation is necessary. However, the chitosan obtained requires an additional purification step by reprecipitation with a solution in acetic acid. The chitin–melanin complex is insoluble in organic and mineral acids, making it harder than pure chitin. Chitin extracted using phosphoric acid is highly amorphous, so it is destroyed if deacetylated [51].

The chitin content is known in the larvae, prepupae, pupae, sheddings, cocoons, and fly stages. For the extraction and purification of chitin, the sample of each of the instars was dried and ground, demineralized with HCl, and deproteinized with NaOH until the absence of color and finally washed with demineralized water. The black soldier fly chitin samples were identified as α-chitin. Following the method based on the measurement of glucosamine, sheddings contain 75.7%, pupae 93.9%, prepupae 94.5%, flies 95.7%, larvae 96.3%, and the highest value was found in cocoons with 96.8%. Chitin from prepupae and cocoons was more crystalline than chitin from sheddings, larvae, and flies, with a crystallinity index of 94% compared to 89% [35].

Another study extracted chitin from different stages of black soldier fly development following the method of drying, grinding, demineralizing, and deproteinizing, but there were some modifications with HCl and NaClO at the end. They report a significant difference, though one less than that reported in other works, in the chitin content of larvae (3.6%), prepupae (3.1%), cocoons (14.1%), and flies (2.9%), indicating that chitin belongs to α-chitin. The crystallinity of chitin at different stages of development showed some differences [52]; it can be seen that the highest chitin content was in the cocoon stage regardless of the isolation method used.

The use of larval prepupae fed with fruit waste has been reported for the extraction of chitin and chitosan. For both extractions, the same demineralization and deproteinization procedure was carried out; however, the prepupae were previously boiled for 15 min. In total, 18.05% of dark-colored chitin and 10.85% of white chitosan were obtained due to it being previously subjected to a depigmentation process [53].

Hahn et al. [54] used cocoons of black soldier fly larva prepupa fed with waste from the plant food processing industry; formic acid was used as an organic alternative for deproteinization. Finally, chitin with around 85% purity was obtained. The extracted α-chitin had a degree of acetylation of 96% and a crystallinity index of 74%.

It is known that for the extraction and purification of chitin and chitosan, reagents that generate toxic waste are used, which is why alternative environmentally friendly methods have been used, such as microbial fermentation methods. Lin et al. [39] used cocoons from larvae fed with soybean curd residues, which were dried and ground after the extraction. *Bacillus lichenformis* was used for DP; subsequently, for DC, sodium bicarbonate and H_2_O_2_ were finally deacetylated with an alkaline solution of sodium hydroxide. An α-chitin-type chitin with an amorphous and non-porous structure was obtained. It was found that the recovery rate of the chitin content was 12.4%, and the degree of deacetylation of the chitosan was 81.5%.

One study compared the enzymatic extraction method versus the chemical method with additional mechanochemical milling and ultrasonication pretreatment. The prepupae of black soldier fly larvae fed with fruit and vegetable waste were used, and shrimp shells were used in comparison. Chemical extraction with ultrasonication resulted in a greater elimination of proteins, obtaining the highest value of α-chitin extraction at 79.9%, though this was not significant with the rest of the chemical extractions; however, it was superior to the enzymatic extraction with 47.6%. The chitin content in shrimp was higher with 88.3%. From a structural outlook, fly chitin has a lower crystallinity than crustacean chitin and a different surface morphology. Although insect chitin has similar applications to shrimp chitin, insects have greater sustainability due to their ability to bioconvert agricultural and food waste into high-value products [17].

Chitin has been extracted from cocoon and adult flies fed with bran and wheat flour using a biological treatment. To achieve this, it was demineralized and deproteinized following the conventional chemical method. *Bacillus subtilis* and *Acetobacter pasteurianus* were used to extract chitin by fermentation to eliminate proteins and minerals. The extraction of chitin, identified as α-chitin, by the biological method achieved 59.9% and 47.31%, and lower values were reached with the chemical treatment, at 23.82% and 11.99% for cocoon and adult fly, respectively [38]. Table 2 shows the crystallinity index, the degree of acetylation, the chitin content, and the method of isolation in the different stages of the black soldier fly’s growth, from the larval to the adult stage.

## 6. Use of Biopolymers in Concrete

Biopolymers enhance the compressive strength of cement concrete by acting as fillers, which densify the concrete matrix, lower porosity, and strengthen interparticle bonding. This leads to greater compressive strength and better load-bearing capacity. Additionally, the inclusion of biopolymers boosts the flexural strength of cement concrete. As reinforcing agents, they improve the material’s resistance to bending and tensile forces, thereby enhancing overall structural integrity [55]. Organic waste generated by industries is considered a renewable biological resource that can be used as an input to produce various value-added products [40]. Recovered biopolymers such as chitin and chitosan are highly important since they can be used as an additive in concrete.

Cactus extract biopolymer has been shown to improve the viscosity of the concrete mix and thus its workability, mechanical properties, and durability characteristics. The setting time for biopolymer-modified cement; the properties of concrete; slump values, split tensile strength, Young’s modulus, and dynamic modulus; and the compressive and flexural strength increased when increasing the amount of cactus extract from 2% to 10% [56].

Alginate, a biopolymer derived from large brown seaweeds, has been studied for its effects when added to concrete in varying percentages (0%, 0.5%, 1%, and 1.5% of cement weight). The findings indicated that incorporating alginate positively influenced the fresh properties of concrete. Notably, increasing the percentage of alginate improved the slump and fresh density, with the 1.5% addition showing the most significant enhancements, 2.5% for fresh density, and 41% for slump compared to the reference mix without additives. Additionally, the hardened properties exhibited improvements, with the highest increases in compressive, splitting tensile, and flexural strength observed at 0.5% and 1.5% alginate additions, yielding an approximate increase of 40% [57].

Arabic gum biopolymer (AGB) has shown significant potential as a water-reducing admixture in concrete, improving its physical and mechanical properties and durability. The addition of AGB resulted in an 8% increase in compressive strength, reaching 41 MPa, while the ultrasonic pulse velocity (UPV) values rose by approximately 40%. Furthermore, concrete with AGB exhibited more excellent resistance to acid attack than plain concrete. X-ray diffraction analysis was conducted to elucidate the impact of AGB on the macroscopic behavior of the concrete [58].

### 6.1. Chitin

Incorporating functionalized chitin nanowhiskers (CTNWs) yields improvements in concrete’s fluidity and compressive strength. A 43% increase in fluidity is observed with the addition of 0.5% functionalized CTNWs, with a slight improvement at higher concentrations. Additionally, a 22% increase in compressive strength is recorded in 28-day concrete samples with the addition of 0.5% CTNWs [59].

A cement paste has been prepared by adding chitin derived from shrimp shells in the form of nanocrystals produced by oxidation and nanofibers produced by the mechanical fibrillation method. With nanocrystals, the setting time of the cement increases, and the nanofibers delay setting; the consistency of the fresh cement paste with both forms of nanochitin is slightly greater than cement without additives. The compressive strength increased, presenting its maximum capacity at 28 days with 5% nanocrystals and 12% nanofibers. The flexural strengths of both nanochitins also improved [33].

### 6.2. Chitosan

Chitosan has hydroxyl (–OH) and amine (–NH_2_) groups [21], which can form hydrogen bonds with the silanol (–Si–OH) groups present in hydrated cement phases like calcium silicate hydrate (C–S–H). These bonds contribute to the adhesion of the particles, thus creating a material of greater strength [60].

Nisticò et al. [61] demonstrated that the use of chitosan, derived from biological waste from the shellfish industry, and its carbon, obtained by pyrolysis at 800 °C, as fillers in a mixture with cement and water, reduced the flexural strength by 25.8% for the case of pure chitosan and 30.5% in the case of carbon compared to concrete without fillers. Likewise, there was a reduction in compression strength of 2.5% for the mixture with chitosan and 24.1% for the case of carbon; however, there was an increase in toughness of 16.1% for the cement and chitosan mixture and 17.7% for the cement and carbon mixture.

Catechol-functionalized chitosan (Cat–Chit), a bioinspired polymer that mimics the structures and functions of natural biological materials, has been synthesized and blended with cement mortar in varying proportions by Choi et al. [62]. The resulting mixes containing 7.5% Cat–Chit polymer in water (CPW) achieved the highest compressive strength, approximately 20.2% greater than the control sample without the polymer. Additionally, the tensile strength of samples with 5% or more CPW was 2.3% to 11.5% higher than the control. Overall, incorporating Cat–Chit effectively enhanced the cement mortar’s mechanical properties, carbonation resistance, and chloride-ion penetration resistance.

Choi et al. [63] explored the potential of a chitosan-based polymer (CBP), a biomimetic polymer, for use in cement mortar with steel slag as a fine aggregate. The CBP was synthesized through an amide coupling reaction involving chitosan, 1-ethyl-3-(3-dimethylaminopropyl) carbodiimide hydrochloride, and 3-(3,4-dihydroxyphenyl) propionic acid. When added to cement mortar containing natural sand or blast furnace slag aggregates, the CBP enhanced both the compressive and tensile strength. However, in mixes with ferronickel slag aggregate, the tensile strength decreased by approximately 5.7% to 25.4% with the addition of CBP. Additionally, the CBP reduced the total charge passed through the mixes, particularly improving the chloride-ion penetration resistance in the mortar using steel slag aggregate. Overall, the findings indicate that the prepared CBP is an effective enhancing agent and shows promising compatibility with cement composites that include steel slag aggregates.

Another study used 0.05% chitosan from shrimp shell waste as an additive in the production of concrete, using a mixture of concrete made with cement, sand, crushed stone, and water as a control. The extraction of chitosan consisted of washing, drying, and grinding the shrimp waste, then demineralization with 4% HCl, and subsequently deproteinization with 5% NaOH until chitin was obtained; finally, it was deacetylated by adding 70% NaOH. The tests showed that the slump value decreased by 14.29%, the density of the concrete improved by 0.86%, and the compressive strength increased by 18.14% (after seven days) and 6.51% (after twenty-eight days) [64].

Baykara et al. [60] mixed chitosan particles from shrimp shells with high early strength hydraulic cement at varying percentages (0, 0.05, 0.25, 0.5, 1, and 2 wt%) and silica sand to create mortar samples. The results indicate that chitosan influences the hydration process by altering the distribution of chitosan particles within the mortar matrix, leading to slight improvements in the midterm mechanical properties. However, incorporating chitosan does not significantly affect the resistance or mechanical properties of the composite. Additionally, the presence of chitosan particles seems to slow down the hydration of the cement mortar.

Citric acid-modified chitosan (CAMC) acts as a thickening agent and extends the setting time of concrete. While CAMC negatively affects the early strength of concrete, it positively contributes to strength development over time. As the CAMC content increased, the self-shrinkage rate of the concrete samples decreased from 86.82 to 14.52 με. Additionally, the long-term drying shrinkage rate dropped from 551.46 to 401.94 με. Furthermore, low doses of CAMC significantly enhanced the impermeability and density of the concrete, improving its resistance to freeze–thaw cycles [65]. Table 3 shows a comparison of the mechanical properties of chitin and chitosan. Table 4 lists the main advantages and disadvantages of using some biopolymers in manufacturing concrete in comparison to simple concrete as a control.

## 7. Conclusions

Chitin and chitosan production from black soldier flies offers an economically viable and environmentally friendly solution, especially given these insects’ rapid growth and high biomass yield. Several companies are investing in commercializing insect-derived chitosan, indicating promising market potential. The physical and chemical properties of chitin and chitosan from different parts of the black soldier fly are similar to those of shrimp waste, which shows that they could be used in the elaboration of cementitious materials to improve their physicochemical and rheological properties for use in the construction industry.

While crustaceans offer a higher biomass volume for chitin and chitosan extraction but lower or equal yields, their processing can be environmentally detrimental. Unlike crustaceans, which require aquatic environments and specialized farming conditions, the black soldier fly presents an environmentally friendly alternative with a short life cycle and the sustainable cultivation of organic waste. This makes it a promising source for chitin and chitosan production in industrial applications.

Chemical extraction, which involves acids and alkalis, has a high carbon footprint, significant energy consumption, and generates large amounts of hazardous waste. Bio-based extraction using microorganisms offers a more sustainable alternative, but its efficiency can be lower. It is the most eco-friendly option, significantly reducing energy use and waste production while minimizing toxic byproducts. The lower environmental burden of bio-based extraction methods supports the sustainability of black soldier fly-derived chitin and chitosan, making it a viable and responsible choice for industrial applications.

Future studies should focus on evaluating these biopolymers as sustainable additives in ferrocement, coatings, blocks, mortars, and composites, contributing to improved mechanical properties and durability. Comparative studies of chitin and chitosan extracted by different methods and their effect on construction materials are also encouraged.

## Figures and Tables

**Table 1:** Comparison of chitin and chitosan extraction methods.

Method	Process Description	Advantages	Disadvantages	References
**Chemical**	Acid and alkali treatment for demineralization, deproteinization, and deacetylation	High-efficiency and fast process	Degradation of polymer structure, harsh chemicals, and environmental concerns	[14,34,35,36]
**Bio-based**	Biological use of microorganisms (bacteria or fungi) for demineralization and deproteinization.	It is an eco-friendly, mild process and retains polymer quality	Slower process and variability in yield	[37,38,39]
	Enzyme-based deproteinization using proteases (e.g., papain, trypsin)	Selective reaction and preserve polymer integrity	It is expensive and requires optimization for large-scale use	[17,41]

**Table 2:** Chitin content, isolation method, degree of deacetylation, and crystallinity index in different growth stages of the black soldier fly.

Growth Stage	Isolation Method	Chitin Content (%)	Degree Deacetylation (DDA%)	Crystallinity Index (%)	Reference
Larva	Chemical	96.3	92	89	[35]
	Chemical	84.0	92	84	[28]
	Chemical	3.6	-	33.09	[52]
Prepupa	Chemical	94.5	77.9	94	[35]
	Chemical	79.9	-	-	[17]
	Chemical	18.05	-	-	[53]
	Chemical	3.1	-	35.14	[52]
	Enzymatic	47.6	-	27	[17]
Pupa	Chemical	93.9	96.7	93	[35]
Sheddings	Chemical	75.7	93.4	90	[35]
Cocoons	Chemical	96.8	89.8	94	[35]
	Chemical	86.8	90	62	[28]
	Chemical	23.82	-	-	[38]
	Chemical	14.1	-	68.44	[52]
	Chemical/organic	85	96	74	[54]
	Biological	59.9	18.52	-	[38]
	Biological	12.4	81.5	52.8	[39]
Adult fly	Chemical	95.7	-	89	[35]
	Chemical	85.3	93	93	[28]
	Chemical	11.99	-	-	[38]
	Chemical	2.9	-	87.92	[52]
	Biological	47.31	37.38	-	[38]

**Table 3:** Comparison of mechanical properties of chitin and chitosan.

Property	Chitin	Chitosan	References
**Compressive Strength**	High	Low	[32,57,64]
**Tensile Strength (MPa)**	Higher due to crystallinity	Lower than chitin; it depends on the degree of deacetylation	[61,62,65]
**Elasticity**	Brittle and rigid	More flexible compared to chitin	[56,59,63]
**Solubility**	Insoluble in water and most solvents	Soluble in acidic solutions (pH < 6.5)	[58,60]
**Biodegradability**	Slow	Faster than chitin	[39]

**Table 4:** Advantages and disadvantages of biopolymer in concrete.

Biopolymer	Source	Advantages	Disadvantages	Reference
-	Cactus (*Opuntia Ficus Indica*)	Chemical-free mechanical extraction. The additive lowers the water demand of the concrete. The compressive and flexural strength increases up to 40% and 37%, respectively, after 90 days of curing. Natural additives, as substitutes for chemical additives, are eco-friendly and cost-effective.	The additive is extracted from a material that can be used primarily as a foodstuff.	[56]
Alginate	-	A 1% alginate addition increases compressive, tensile, and flexural strength by 11%, 5.3%, and 3.4%, respectively.	Biopolymer from a commercial brand by chemical extraction	[57]
Arabic gum	-	The highest compressive strength is observed in the concrete containing 1.1% biopolymer. Its inclusion enhances the acid resistance of the concrete.	Arabic gum harvesting may cause a negative environmental impact.	[58]
Chitin nanowhiskers	-	Adding 0.75% of biopolymer increases the compressive strength and flowability, 31% and 46.9%, respectively, higher than simple concrete.	Biopolymer from a commercial brand by chemical extraction.	[59]
Chitin	Shrimp shells	Biopolymer obtained from organic wastes. Adding 0.05 wt% biopolymer provides the longest setting time compared to mortars containing higher and lower biopolymer concentrations. The most significant improvement of 2.5% in compressive strength is recorded with 0.02% chitin at 28 days. At 28 days, the modulus of elasticity increased by 91% compared to simple concrete with 0.02 wt% chitin. At 28 days, all chitin doses show an improvement ranging from 7% to 9%.	Chemical extraction.	[33]
Chitosan	Shellfish	Biopolymer obtained from organic wastes. A 16% increase in toughness with chitosan addition compared to plain concrete. Compressive strength is similar to simple concrete.	Chemical and energy-intensive extractions. Lower flexural strength compared to simple cement.	[61]
Chitosan	-	Adding chitosan does not significantly degrade the fluidity of the cement mortar. At 28 days, the compressive strength increased by 20% compared to simple concrete with 7.5% chitosan. The highest tensile strength is achieved by adding 10% chitosan, increasing by 11.5% concerning simple concrete.	Biopolymer from a commercial brand by chemical extraction.	[62]
Chitosan	-	At 56 days, the compressive strength increased by 13% compared to simple concrete with 10% chitosan. At 28 days, the split tensile strength increased by 20% compared to simple concrete with 10% chitosan.	Biopolymer from a commercial brand by chemical extraction.	[63]
Chitosan	Shrimp shells	Seafood waste valorization. Concrete cost reduction. Adding 0.05 wt% chitosan increases compressive strength, indirect tensile strength, and flexural strength by 6.51%, 33.33%, and 40.9% after twenty-eight days, respectively, compared with simple concrete.	Chemical extraction. The slump value decreases by 14.29% compared to simple concrete.	[64]
Chitosan	Shrimp shells	Seafood waste valorization. Adding 0.25 wt% chitosan increases compressive strength by 4% after 28 days compared with simple concrete.	Chemical extraction. The climate change score for 0.25% wt was 1.3% higher compared to simple concrete.	[60]
Chitosan	-	Adding 0.6% mass chitosan increases compressive strength by 21.4% after 28 days compared with simple concrete. Adding 0.4% mass chitosan increases splitting tensile strength by 9.6% after 28 days compared with simple concrete.	Chemical extraction. As the chitosan content increases, the fresh mixing performance of the concrete is affected.	[65]

## Data Availability

Data are contained within the article.
